# BRD4 PROTAC degrader enhances fulvestrant sensitivity in ER+ breast cancer via super-enhancer associated GREB1

**DOI:** 10.3389/fonc.2026.1828900

**Published:** 2026-05-19

**Authors:** Xiulei Zhang, Peiming Zheng, Zhengyi Liu, Qian Zhang, Wenjing Duan, Tingting Zhang, Xiaozhuan Liu

**Affiliations:** 1 Department of Central Laboratory, Henan Provincial People’s Hospital, Zhengzhou University, Zhengzhou, China; 2 Department of Clinical Laboratory, Henan Provincial People’s Hospital, Zhengzhou University, Zhengzhou, China; 3 Department of Breast Surgery, Henan Provincial People’s Hospital, Zhengzhou University, Zhengzhou, China; 4 Henan Provincial Key Medical Laboratory of Genetics, Zhengzhou University, Zhengzhou, China

**Keywords:** BRD4, breast cancer, fulvestrant sensitivity, GREB1, PROTAC

## Abstract

**Background:**

Breast cancer has the highest incidence and mortality among all cancers affecting women. Fulvestrant resistance remains a major clinical challenge that limits the efficacy of endocrine therapies. BRD4, a transcriptional regulator that recognizes acetylated histones, is implicated in the pathogenesis and progression of various tumors, including breast cancer. However, its role in fulvestrant sensitivity and the therapeutic potential of targeted BRD4 degradation require further investigation.

**Methods:**

We assessed BRD4 transcriptional activity in breast cancer and its functional role in tumor progression and endocrine sensitivity. The antitumor effect of a PROTAC-targeted BRD4 degrader, alone or in combination with fulvestrant, was evaluated in breast cancer cells. Integrated analysis of BRD4 and estrogen receptor (ER) chromatin immunoprecipitation sequencing (ChIP-seq) datasets was performed to identify co-occupied genomic regions and downstream targets. GREB1 was identified as a key effector and further validated as a super-enhancer-associated gene. The working mechanism of BRD4 PROTAC and fulvestrant was investigated through GREB1 signaling disruption.

**Results:**

The occupancy of BRD4 at promoter regions was found to be increased in breast cancer, and its high expression indicated poor clinical outcome among ER+ breast cancer patients with endocrine therapy. A PROTAC-targeted BRD4 degrader significantly enhanced the antitumor efficacy of fulvestrant in breast cancer cells. Integrated ChIP-seq analysis revealed substantial co-occupancy of BRD4 and ER on shared pathways and identified GREB1 as a critical downstream effector regulated by a BRD4-associated super-enhancer. Mechanistically, the BRD4 PROTAC enhances fulvestrant sensitivity by down-regulation of GREB1 expression.

**Conclusion:**

Targeting BRD4 with PROTAC degraders represents a promising therapeutic strategy in breast cancer by suppressing GREB1 expression and enhancing the efficacy of fulvestrant.

## 1 Introduction

Breast cancer, particularly the estrogen receptor-positive (ERα+) subtype, relies on the transcriptional activity of ER for its progression. While endocrine therapies that target this pathway are the cornerstone of treatment, the emergence of resistance remains a predominant clinical challenge (1, 2). The bromodomain and extraterminal (BET) protein BRD4, a key epigenetic reader of histone acetylation, has emerged as a critical regulator of oncogenic transcription in various cancers, including breast cancer (3). It facilitates transcriptional elongation by recruiting positive transcription elongation factor b (P-TEFb) and other regulators to super-enhancers (4). Critically, BRD4 has been shown to co-operate with ER in driving the transcriptional programs that support breast cancer cell growth and survival (5). However, the specific mechanism of this synergy, especially its role in fostering endocrine sensitivity, is not fully elucidated.

Our data and other reports suggest that BRD4 and ER co-occupy a significant number of genomic loci, pointing to a shared transcriptional network. Furthermore, the growth regulating estrogen receptor binding 1 (GREB1), a direct ER target critical for hormone-dependent growth, is hypothesized to be a central node in this network (6). We postulated that BRD4 is indispensable for the robust expression of GREB1 and other shared target genes, and that its increased promoter occupancy may contribute to endocrine resistance. We employed an integrated multi-omics approach combining ChIP-seq and RNA-seq analyses in MCF7 breast cancer cells. We defined the overlapping genomic landscapes of BRD4 and ER, confirming their extensive co-localization. We then demonstrated that the BRD4-targeting PROTAC degrader ZBC260 enhances fulvestrant sensitivity. We further identified GREB1 as a key super-enhancer-associated gene co-regulated by BRD4 and ER. Our findings establish that targeted degradation of BRD4 disrupts the GREB1-centered transcriptional program, thereby suppressing cell proliferation and migration. This work provides a compelling rationale for co-targeting BRD4 and ER as a novel therapeutic strategy to enhance endocrine-sensitivity in breast cancer.

## 2 Materials and methods

### 2.1 Cell culture

MCF7 cells were cultured in DMEM (BI, Israel) supplemented with 10% fetal bovine serum (FBS; BI, Israel) and 1% penicillin-streptomycin (Solarbio, China) and were authenticated by short tandem repeat (STR) profiling (Genewiz, China).

### 2.2 ChIP-seq data analysis

ChIP-seq datasets were obtained from the GEO database. Raw reads were first assessed for quality using FastQC (10) and then aligned to the hg38 reference genome using Bowtie2 (11). The resulting SAM files were filtered (MAPQ ≥ 30), merged, and converted to BAM format using SAMtools (12). Peak calling was performed with MACS2 under default parameters (13), and the called peaks were annotated with the ChIPseeker package against the hg38 genome annotation (14). Finally, binding density profiles around the transcription start site (TSS ± 3 kb) were generated and visualized with deepTools (15).

### 2.3 RNA-seq data analysis

RNA-seq datasets were acquired from the GEO database. Raw reads were first subjected to quality control using FastQC (10), and adapter sequences along with low-quality bases were trimmed using Cutadapt (16). The cleaned reads were then aligned to the hg38 reference genome using HISAT2 (17). The resulting SAM files were filtered (MAPQ ≥ 30) and sorted using SAMtools (12). Read counting for genes was performed with HTSeq (18), followed by differential expression analysis using DESeq2 (19), with a significance threshold set at |log_2_FC| ≥ 1 and an adjusted *p-value* < 0.05.

### 2.4 Function enrichment analysis

KEGG pathway enrichment was conducted using the clusterProfiler package with a significance cutoff of *p < 0.05* (20). Specifically, genes associated with differential binding peaks in promoter regions (TSS ± 3 kb) from ChIP-seq data, along with differentially expressed genes from RNA-seq data, were subjected to separate enrichment analyses.

### 2.5 Super enhancer associated genes

Peaks of BRD4 and H3K27ac were identified *via* the MACS2 algorithm to define constituent enhancers, which were stitched together within a 12.5 kb window using the ROSE algorithm to identify super-enhancers. Enhancer regions were visualized using hockey stick plots with enhancers ranked by H3K27ac or BRD4 signal intensity. Enhancers above the inflection point of the curve were defined as super-enhancers (BRD4 cut-off, 8034.7455). Annotation of enhancers and super-enhancers were also performed using the ROSE package with the hg38 genome annotation (21).

### 2.6 Survival analysis

The prognostic significance of candidate genes was analyzed *via* the KMplot online tool (https://kmplot.com/) (22). The patient cohort was defined as breast cancer cases, ERα-positive breast cancer cases and ERα-positive breast cancer cases treated with endocrine therapy only. Statistical significance was determined by a *p-value < 0.05*.

### 2.7 RT-qPCR validation

Total RNA was extracted using TRIzol reagent (Vazyme, China) according to the manufacturer’s protocol. cDNA was synthesized, and quantitative PCR (qPCR) was performed using RT-qPCR kit (Vazyme, China). Gene-specific primers were designed with Primer3 and the NCBI BLAST tool, and their specificity was confirmed by melt-curve analysis on a StepOne Plus Real-Time PCR System (Invitrogen, USA). The amplification conditions were set as previously described (23). The relative expression levels were calculated using the 2^(-ΔΔCt) method, with 18S rRNA serving as the endogenous control. Statistical significance was determined using a two-tailed paired t-test in GraphPad Prism, with a *p-value < 0.05* considered significant. Primer sequences (5’ to 3’) used in this study were as follows:

18S: Forward-GTAACCCGTTGAACCCCATT, Reverse-CCATCCAATCGGTAGTAGCG;
GREB1: Forward-ATGGGAAATTCTTACGCTGGAC, Reverse-CACTCGGCTACCACCTTCT.

### 2.8 RNA interference

Cells were transfected with si-GREB1 (50nM) (target sequence 5’-CTGGCCGCGGACCAGGTGCCC-3’) (Ribobio, China) using jetPRIME (Polyplus-transfection, France) according to the manufacturer’s instructions.

### 2.9 Immunofluorescence

After washing with PBS, cells were fixed in 4% paraformaldehyde (15 min, RT), permeabilized with 0.1% Triton X-100 (15 min), and blocked with 5% BSA (1 h). Specimens were incubated with primary antibodies BRD4 (Cat No. ab128874, Abcam, UK), and GREB1 (Cat No. 28699-1-AP, Proteintech, USA) overnight at 4 °C, followed by species-matched fluorescent secondary antibodies (1 h, RT) (Cat No. SA00013-4, Proteintech, USA). Nuclei were visualized with DAPI counterstaining. Imaging was performed using an inverted fluorescence microscope (Olympus, Japan).

### 2.10 Cell growth assay

Cells were grown in a 96 well plate with 3,000 cells per well for an additional 24 h before treatment. The cells were treated with gradient concentrations of 0–200 nM Vehicle, JQ1, ARV825 and ZBC260 (MCE, USA) for 48 hours. After treatment, 10μl of CCK-8 solution (MCE, USA) was added to each well containing 100μl of culture medium, followed by incubation at 37 °C for 4h. The absorbance was measured at 450 nm using a microplate reader (BioTek, USA), and cell viability was calculated as the percentage relative to control wells. Cell numbers were quantified by Countess II Automated Cell Counter (Thermofisher, USA).

### 2.11 Cell migration assay

5×10^4^ cells were plated in a 24-well transwell plate with an 8-μm pore size membrane (Corning, USA). The lower chamber was filled with 600ul of medium supplemented with 20% FBS and the upper chamber was filled with 200ul basal medium. After 48 hours of cell culture, cells were fixed by 4% paraformaldehyde for 20min and stained with 1% crystal violet solution for 1h. Cells on the upper chamber were removed by wiping with a cotton swab. Imaging was performed using an inverted microscope (Olympus, Japan).

### 2.12 Calcein-AM/PI assay

Cell viability was assessed using Calcein-AM/PI double staining kit (Solarbio, China). Briefly, cells were washed twice with 1×Assay Buffer and incubated with 2μM Calcein-AM at 37 °C for 30min in the dark and then 5μM propidium iodide (PI) at 37 °C for 5min in the dark. Imaging was performed using an inverted fluorescence microscope (Olympus, Japan).

### 2.13 ChIP-qPCR experiments

ChIP assays were performed as previously described with modifications (24). Briefly, approximately 10^7^ cells were cross-linked with 1% formaldehyde for 10 min at room temperature, followed by quenching with 0.125 M glycine for 5 min. Chromatin was sheared by sonication to an average size of 200–500 bp. Soluble chromatin fragments were immunoprecipitated with 2 μg of anti-BRD4 antibody (Cat No. ab128874, Abcam, UK) overnight at 4 °C. Immunocomplex were captured using Protein A/G PLUS-Agarose beads (Santa Cruz Biotechnology, USA), followed by sequential washing. DNA was purified using the EasyPure Genomic DNA Kit (TransGene Biotech, China) and analyzed by qPCR. GREB1 ChIP-qPCR primer (5’-3’): Forward-CAGGGGCTCCATGTAAAACGA, Reverse-TGTTTAAAGCTGCCACCAACG.

### 2.14 Western blotting

Cells were lysed using RIPA buffer according to the manufacturer’s protocol. Protein samples of equal quantity were separated by SDS-PAGE and transferred to PVDF membranes (Merck, Germany). After blocking with 5% non-fat milk in TBST for 1 h, membranes were incubated overnight at 4 °C with primary antibody against GAPDH (Cat No. 60004-1-Ig, Proteintech, USA), BRD4 (Cat No. ab128874, Abcam, UK), and GREB1 (Cat No. 28699-1-AP, Proteintech, USA). Following extensive washing, membranes were probed with horseradish peroxidase-conjugated secondary antibody (Cat No. SA00001-1, SA00001-2, Proteintech, USA) for 1 h at room temperature. Protein bands were visualized using ECL detection reagent (Thermo Scientific, USA) and imaged with a chemiluminescence detection system (Bio-Rad, USA).

### 2.15 Statistical analysis

All experiments were performed at least three independent biological replicates (n=3). Data are presented as mean ± Standard Error of the Mean (SEM). Statistical significance was determined using a two-tailed paired t-test in GraphPad Prism 6.0 (GraphPad Software, USA), with a *p-value* < 0.05 considered significant.

### 2.16 ChIP-seq and RNA-seq datasets

## 3 Results

### 3.1 The landscape of BRD4 chromatin binding in MCF10A compared to MCF7 cells

**Figure 1:**
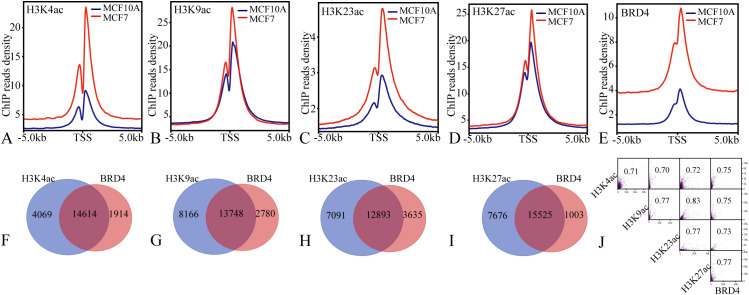
The landscape of BRD4 chromatin binding in MCF10A compared to MCF7 cells. **(A–E)** the H3K4ac, H3K9ac, H3K23ac, H3K27ac and BRD4 chromatin binding landscapes MCF10A and MCF7 cell lines; **(F–J)** the co-localization sites and correlation coefficients between H3K4ac, H3K9ac, H3K23ac, H3K27ac and BRD4 chromatin binding events.

Histone 3 lysine acetylation and its reader protein BRD4 play a pivotal role in the epigenetic regulation of breast cancer. To delineate the epigenetic landscape governed by H3K acetylation and BRD4 in breast cancer tumorigenesis, we analyzed publicly available ChIP-seq datasets for H3K4ac, H3K9ac, H3K23ac, H3K27ac and BRD4 (**Table 1**). Our analysis revealed the chromatin binding landscapes of these epigenetic marks in non-tumorigenic MCF10A and breast cancer MCF7 cell lines. Notably, the chromatin binding densities of all five factors were significantly elevated in promoter regions of MCF7 cells compared to MCF10A cells (**Figures 1A–E**). Furthermore, a substantial majority (≥78%) of BRD4 binding sites coincided with histone acetylation sites, specifically H3K27ac (93.93%), H3K4ac (88.42%), H3K9ac (83.18%), and H3K23ac (78.00%). These co-localization events were supported by strong correlation coefficients, all exceeding 0.7 (**Figures 1F–J**). These findings demonstrate a robust synergistic relationship between histone acetylation and BRD4 occupancy, highlighting their potential as key epigenetic drivers in breast cancer pathogenesis.

**Table 1:** The details of the public available GEO datasets utilized in this study.

MCF10A/MCF7 ChIP-seq	E2 treatment MCF7 ChIP-seq	JQ1 treatment MCF7 RNA-seq
H3K4ac GSE69377	ER GSE167451	GSE123285
H3K9ac GSE85158	P300 GSE29073	
H3K23ac GSE85158	H3K27ac GSE113092	
H3K27ac GSE85158	BRD4 GSE55921, GSE123284	
BRD4 GSE72931, GSE123284	Pol II GSE23701	

### 3.2 High BRD4 expression predicts poor prognosis in breast cancer patients

**Figure 2:**
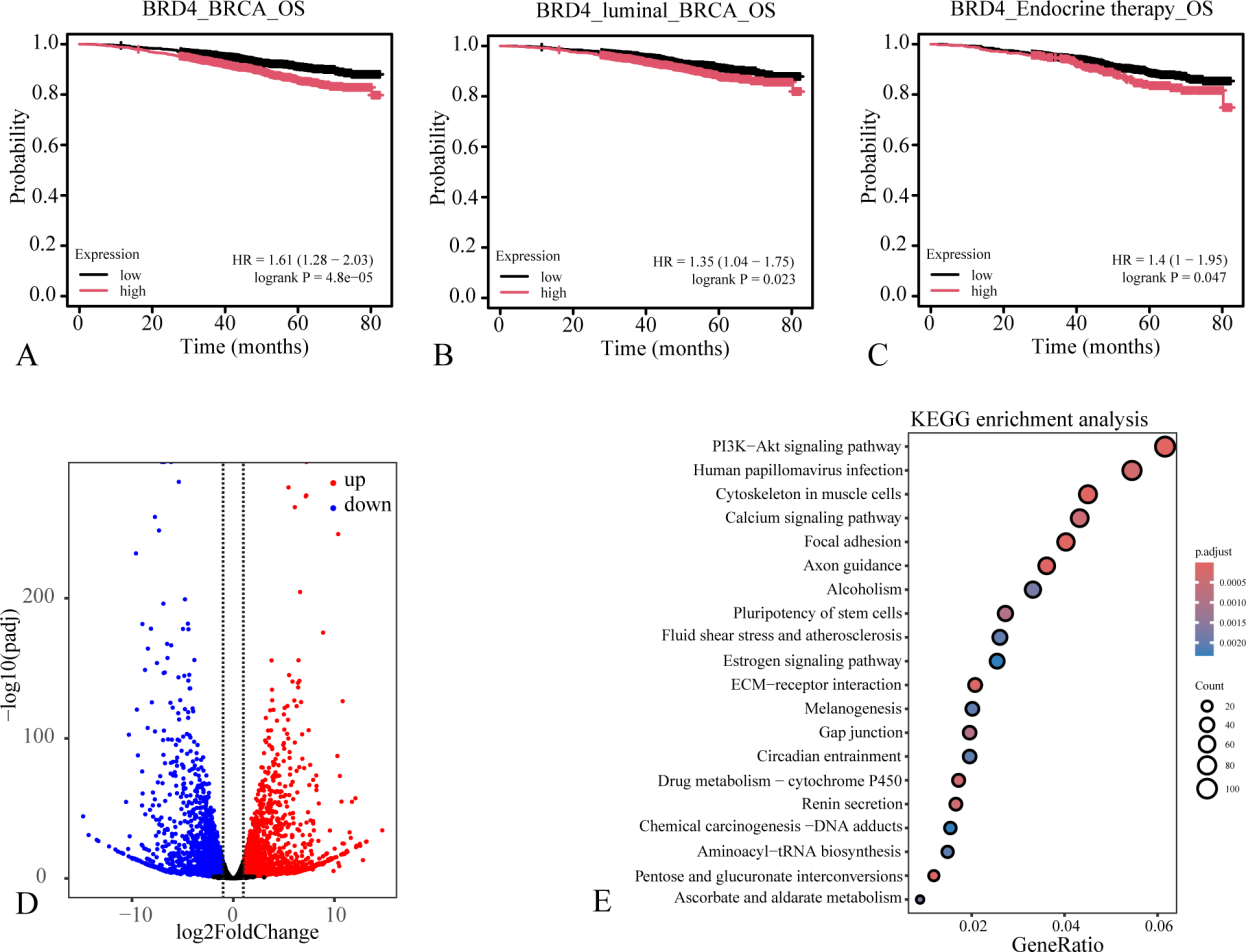
High BRD4 expression predicts poor prognosis in breast cancer patients. **(A–C)** Kaplan-Meier survival analysis of BRD4 in breast cancer patients, luminal subtype breast cancer patients and these patients undergoing endocrine therapy; **(D)** volcano plot of the differential expression genes in MCF7 fulvestrant resistant cell line; **(E)** dot plot of the KEGG pathway enrichment analysis of the differentially expressed genes in MCF7 fulvestrant resistant cell line.

To evaluate the prognostic value of BRD4 in breast cancer, we performed Kaplan-Meier survival analysis. The results demonstrated that a high expression level of BRD4 was significantly associated with reduced survival probability in breast cancer patients, including those with the luminal subtype (**Figures 2A, B**). Furthermore, elevated BRD4 expression also predicted poor prognosis in patients undergoing endocrine therapy (**Figure 2C**). Given these findings, we subsequently analyzed RNA-seq data from parental MCF7 cells and fulvestrant-resistant MCF7 cells (GSE118713). This analysis revealed that BRD4 expression is significantly upregulated in the fulvestrant-resistant cell line (**Figure 2D**, **Supplementary Table 1**). KEGG pathway enrichment analysis of the differentially expressed genes in these resistant cells identified significant enrichment in the PI3K-Akt and estrogen signaling pathways, which were classic signaling pathways in endocrine resistance (**Figure 2E**). Our results establish BRD4 as a critical driver of tumor progression and endocrine resistance, potentially through the modulation of key oncogenic signaling pathways.

### 3.3 Targeted degradation of BRD4 by a PROTAC sensitizes breast cancer cells to the anti-proliferative effects of fulvestrant

**Figure 3:**
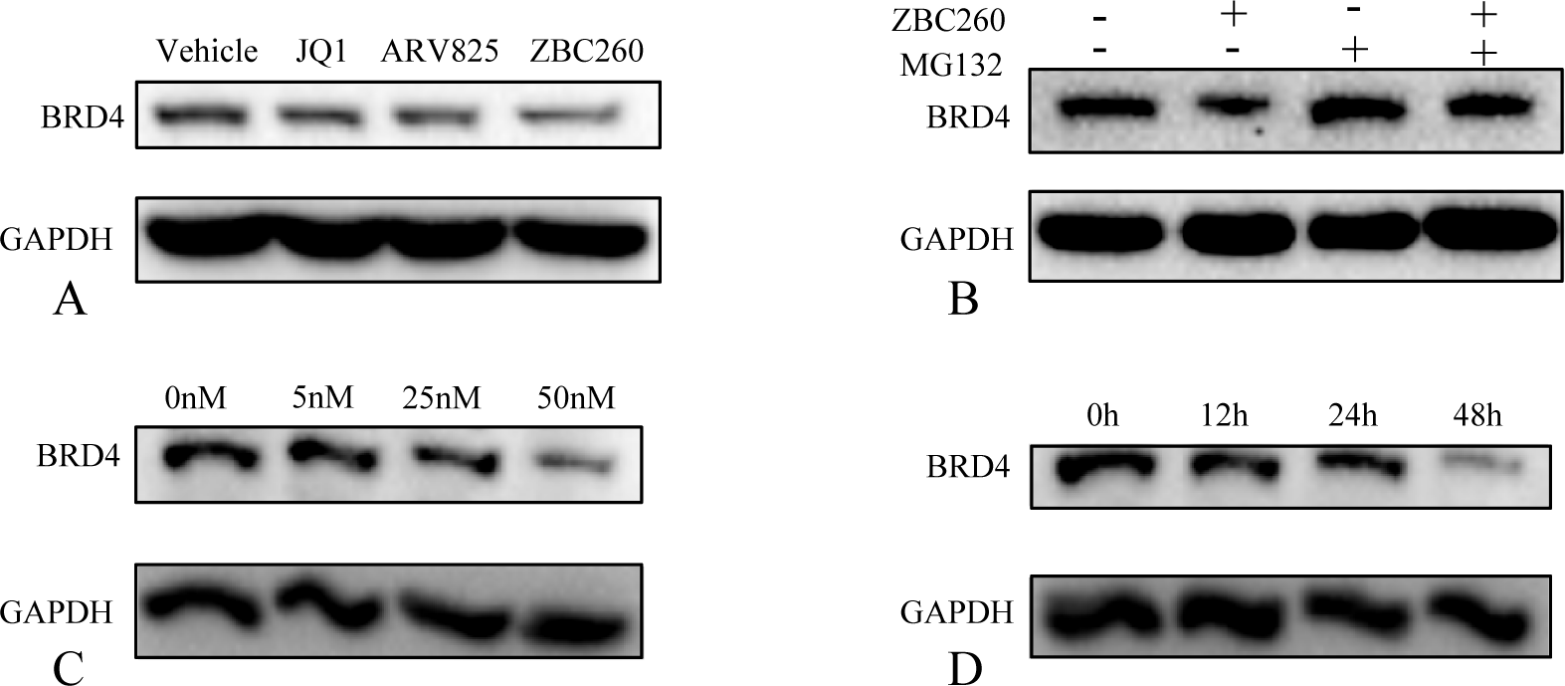
ZBC260 induces potent and proteasome-dependent degradation of BRD4. **(A)** western blot analysis of the protein level of BRD4 after treated with JQ1, ARV825, and ZBC260 (50nM, 48h); **(B)** western blot analysis of the protein level of BRD4 after pre-treated with the proteasome inhibitor MG132 (10μM, 6h), followed with ZBC260 (50nM, 48h); **(C)** western blot analysis of the protein level of BRD4 after treated with gradient concentrations of 0–50nM ZBC260 for 48h; **(D)** western blot analysis of the protein level of BRD4 after treated with 50nM ZBC260 for 0-48hours (n=3).

**Figure 4:**
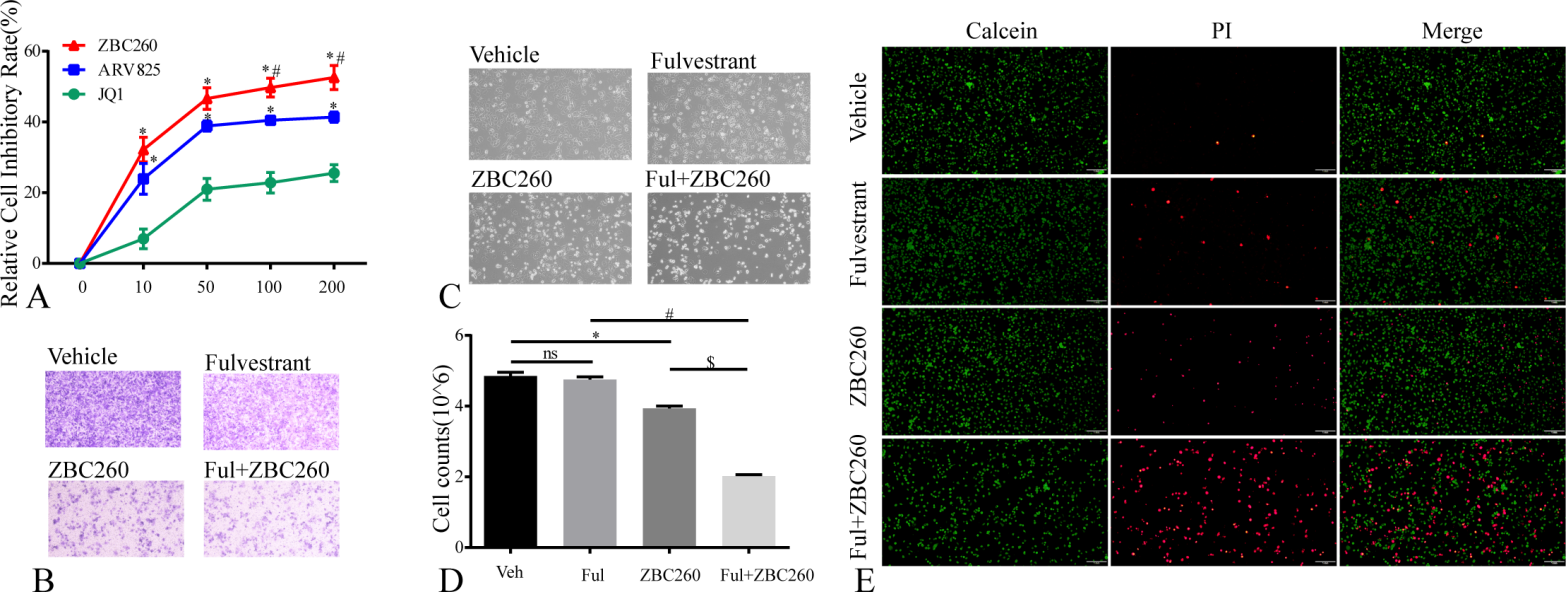
BRD4 PROTAC enhances fulvestrant sensitivity. **(A)** the relative inhibitory rate of JQ1, ARV825 and ZBC260 on MCF7 (0-200nM, 48h); **(B–D)** the representative transwell image, microscope image and cell counts of MCF7 treated with vehicle, fulvestrant (100nM), ZBC260 (50nM) and Ful+ZBC260 for 48h; **(E)** the representative fluorescence microscope image of calcein-AM/PI co-localization staining in MCF7 after separate treatments. * indicates p<0.05, ZBC260 compared to vehicle; # indicates p<0.05, Ful+ZBC260 compared to Ful; $ indicates p<0.05, Ful+ZBC260 compared to ZBC260 (mean ± SEM, n=3).

To evaluate the degradation efficacy of BRD4-targeting PROTACs, we first screened a panel of compounds and identified ZBC260 as the most potent degrader (**Figure 3A**). Furthermore, pre-treatment with the proteasome inhibitor MG132 abrogated ZBC260-induced BRD4 degradation (**Figure 3B**), indicating that ZBC260 promoted BRD4 degradation through the ubiquitin-proteasome pathway. ZBC260 also led to a marked reduction in BRD4 protein levels in a dose- and time-dependent manner (**Figures 3C, D**). In a screen for compounds with anti-proliferative activity, ZBC260 exhibited the strongest inhibitory activity (**Figure 4A**). We further validated that ZBC260 enhanced the inhibitory effect of fulvestrant to more profoundly inhibit cell migration and proliferation and to induce apoptosis (**Figures 4B–E**). BRD4 PROTAC enhanced fulvestrant sensitivity and provided an effective therapeutic strategy against ER+ breast cancer.

### 3.4 The downstream signaling network integrating BRD4 and ER activities

**Figure 5:**
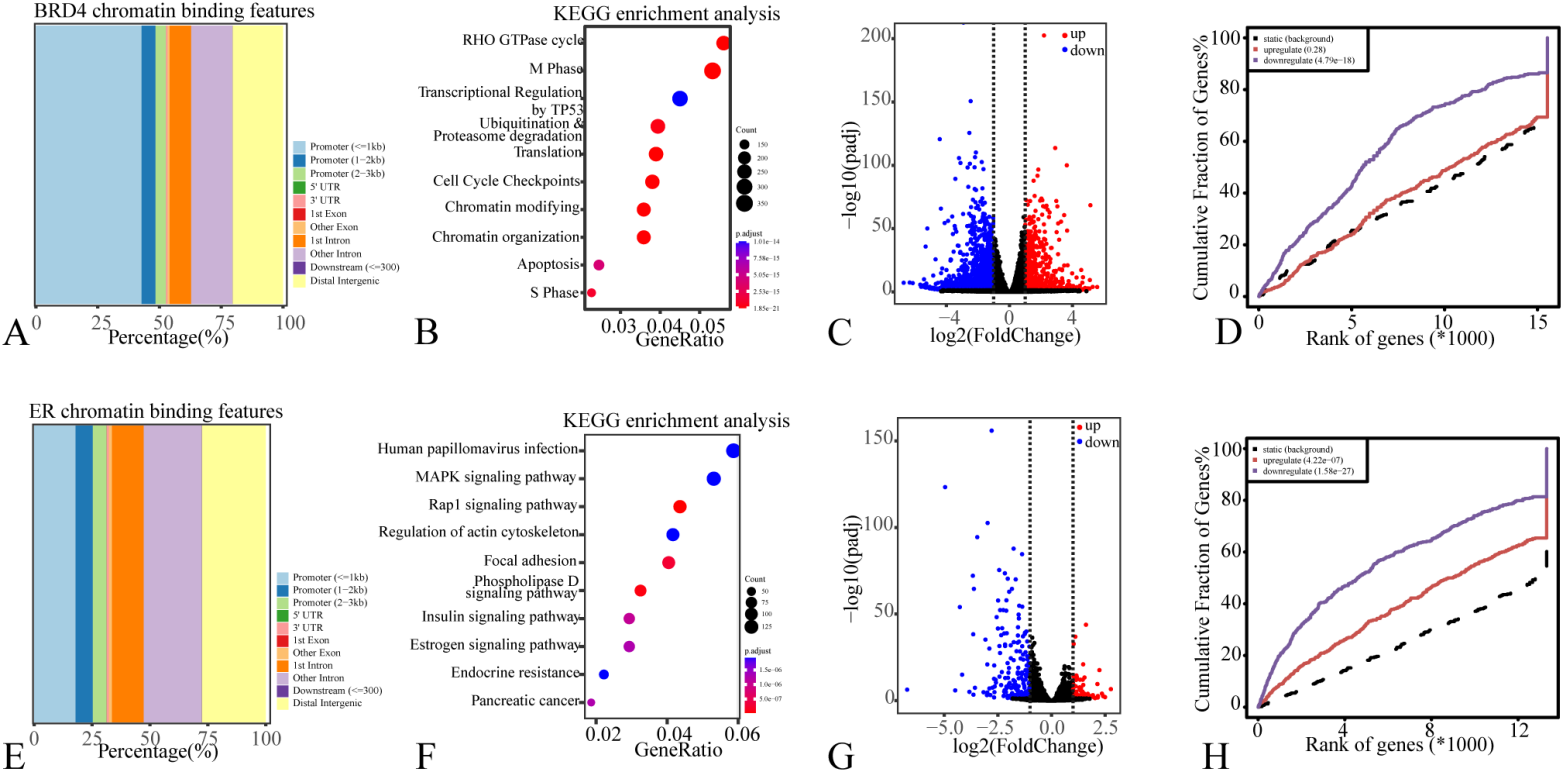
The downstream signaling network integrating BRD4 and ER activities. Bar plot of BRD4 **(A)** and ER **(E)** chromatin binding features; dot plot of the KEGG pathway enrichment analysis of BRD4 **(B)** and ER **(F)** chromatin binding genes; volcano plot of the differential expression genes in MCF7 separately treated with JQ1 **(C)** and fulvestrant **(G)**; curve diagram of cumulative fraction of genes regulated by BRD4 **(D)** and ER **(H)**.

To elucidate the mechanisms by which BRD4 and ER inhibition suppress breast cancer, we conducted an integrated analysis of ChIP-seq and inhibitor-treated RNA-seq data in MCF7 cells. Genomic annotation revealed that approximately 50% of BRD4 binding sites are located in promoter regions (**Figure 5A**). KEGG pathway analysis of genes associated with these promoters indicated that BRD4 primarily regulates processes including transcription, ubiquitination & proteasome degradation, cell cycle checkpoints, and chromatin modification (**Figure 5B**). Consistent with this, differential expression analysis following JQ1 treatment identified a set of genes responsive to BRD4 inhibition (**Figure 5C**). Integration of ChIP-seq and RNA-seq data confirmed that JQ1 predominantly induces a repressive function of BRD4 in MCF7 cells (**Figure 5D**). Parallel profiling of ER binding delineated a distinct but functionally complementary regulatory landscape. About one-third of ER binding sites were found in promoter regions (**Figure 5E**). KEGG enrichment analysis of these promoter-associated genes highlighted ER’s role in the MAPK and RAP1 signaling pathways, focal adhesion, and endocrine resistance (**Figure 5F**). Transcriptomic profiling upon fulvestrant treatment identified ER-regulated genes (**Figure 5G**), and integrated data analysis confirmed that fulvestrant exerts its effect primarily by repressing ER’s transcriptional activity (**Figure 5H**). Taken together, our results map the distinct and cooperative gene networks regulated by BRD4 and ER, revealing a compelling mechanistic basis for synergistic targeting strategies in breast cancer.

### 3.5 The BRD4-targeted PROTAC suppresses GREB1 expression

**Figure 6:**
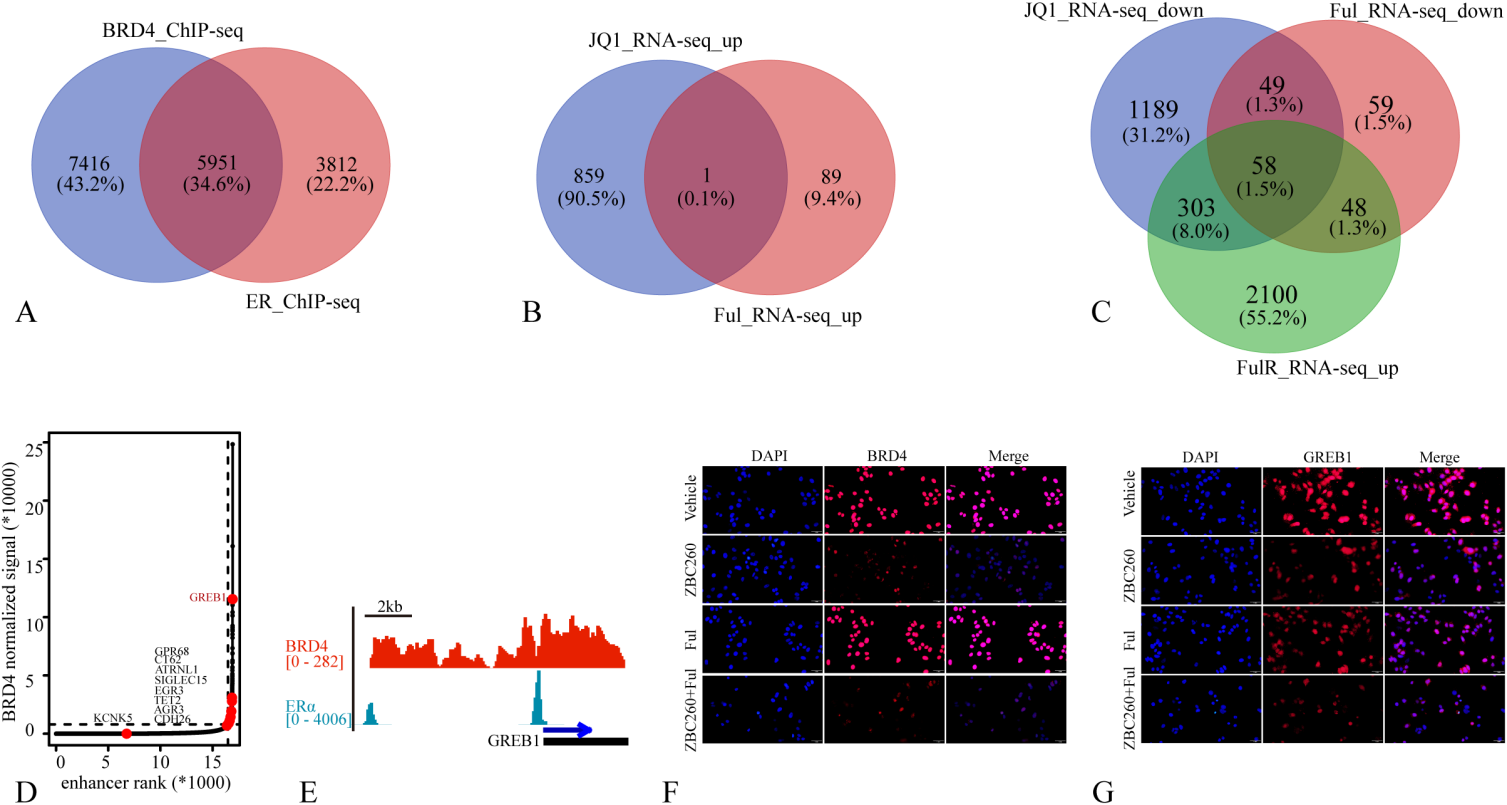
The BRD4-targeted PROTAC suppresses GREB1 expression. **(A)** Venn diagram of BRD4 and ER chromatin binding genes; **(B)** Venn diagram of JQ1 and fulvestrant treatment RNA-seq up-regulated genes; **(C)** Venn diagram of JQ1 and fulvestrant treatment RNA-seq down-regulated genes and fulvestrant resistance up-regulated genes; **(D)** distribution of BRD4 normalized ChIP-seq signal and the corresponding enhancer rank in MCF7 cells; **(E)** the chromatin binding densities of BRD4 and ER on the GREB1 promoter region; **(F, G)** the representative fluorescence microscope image of BRD4 and GREB1 immunofluorescence in MCF7 separately treated with vehicle, fulvestrant(100nM), ZBC260 (50nM) and Ful+ZBC260 for 48h (n=3).

**Figure 7:**
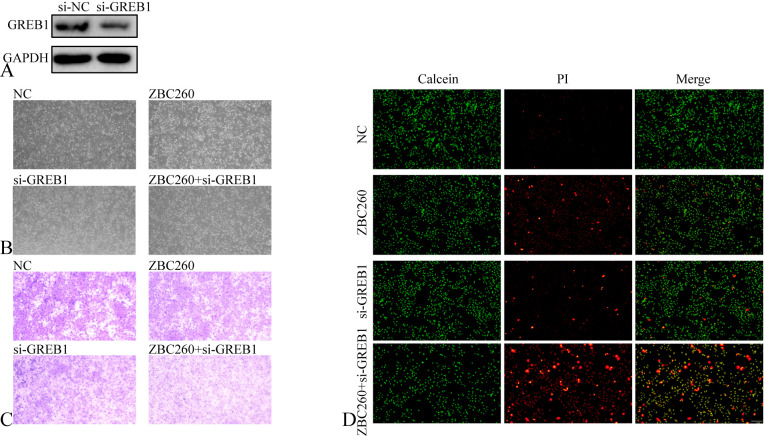
GREB1 knock down enhanced the inhibitory effect of ZBC260. **(A)** western blot analysis of the protein level of GREB1 after treated with GREB1 siRNA (50nM, 48h); **(B, C)** the representative microscope image, and transwell image of MCF7 treated with negative control, ZBC260 (50nM), GREB1 siRNA (50nM), ZBC260 and GREB1 siRNA for 48h; **(D)** the representative fluorescence microscope image of calcein-AM/PI co-localization staining in MCF7 after separate treatments (n=3).

To identify common transcriptional targets of the BRD4 inhibitor ZBC260 and the ER inhibitor fulvestrant, we integrated datasets from BRD4 and ER ChIP-seq (promoter-associated genes) and RNA-seq after JQ1 or fulvestrant treatment. Analysis revealed that 61.0% of ER promoter-binding genes overlapped with BRD4 promoter-binding genes, and 50% of fulvestrant-downregulated genes were also suppressed by JQ1 (**Figures 6A–C**, **Supplementary Table 2**), indicating that the ER signaling pathway shares substantial commonality with that of BRD4. We further intersected the downregulated genes from both JQ1 and fulvestrant treatments with genes upregulated in fulvestrant-resistant cells, identifying 58 candidate genes potentially core to BRD4 and ER function as well as therapy resistance (**Figure 6C**). Given BRD4’s role as a master regulator of super-enhancers (SEs), we mapped its downstream SE landscape and identified 450 SEs (**Supplementary Table 3**). Intriguingly, these SEs were associated with 12 of the 58 shortlisted genes (**Figure 6D**). We focused subsequent validation on GREB1, a key SE-associated gene transcriptionally co-regulated by BRD4 and ER (**Figure 6E**). Immunofluorescence staining in MCF7 cells confirmed that combined ZBC260 and fulvestrant treatment potently inhibited the expression of GREB1 (**Figures 6F, G**). We further validated that GREB1 knock down also enhanced the inhibitory effect of ZBC260 to more profoundly inhibit cell migration and proliferation and to induce apoptosis (**Figures 7A–D**). Our multi-omics integration and experimental validation nominate GREB1 as a critical downstream effector through which BRD4 and ER cooperatively drive breast cancer pathogenesis and therapy sensitivity.

### 3.6 The epigenetic state of GREB1 and it is directly regulated by BRD4

**Figure 8:**
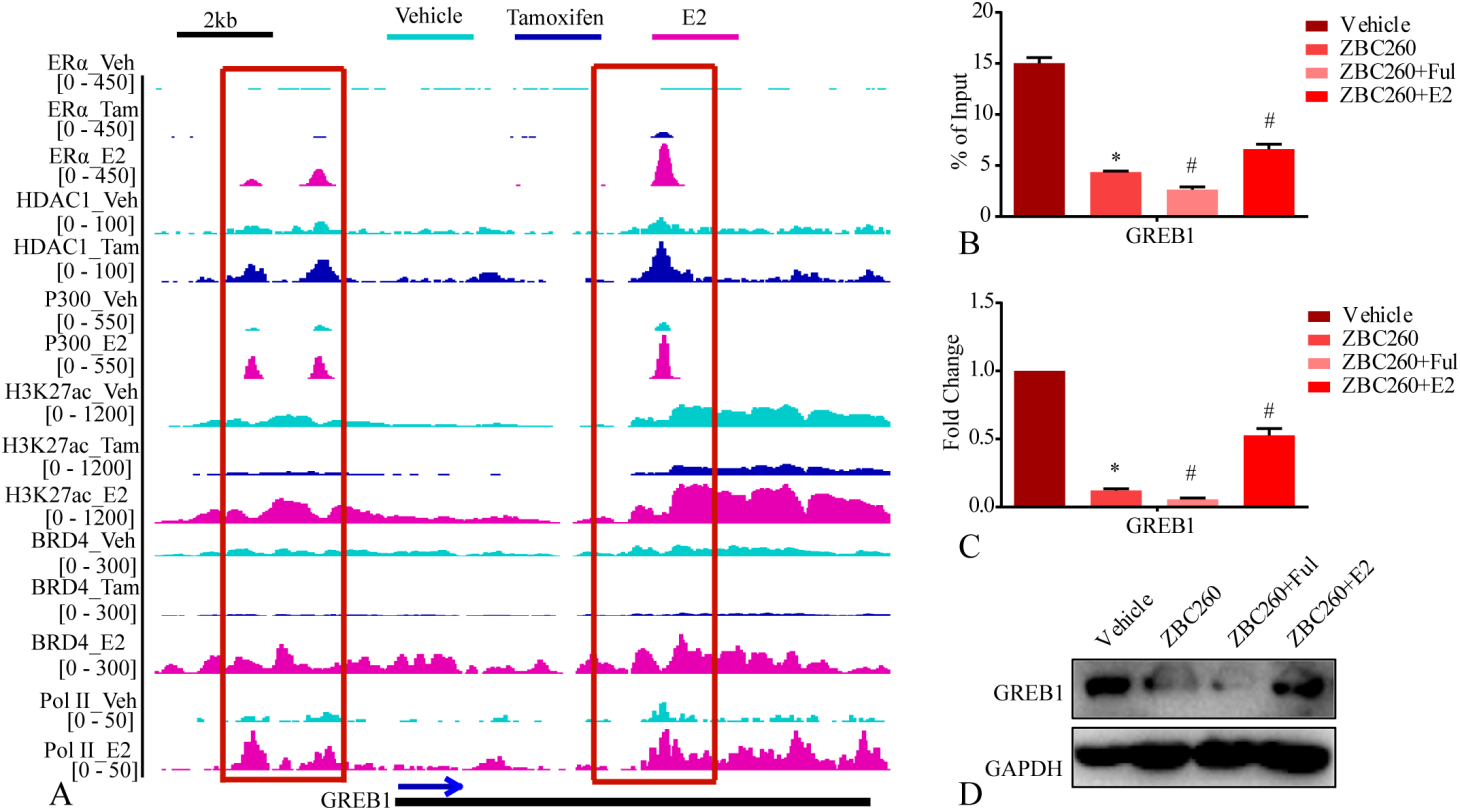
The epigenetic state of GREB1 and it is directly regulated by BRD4. **(A)** the chromatin binding densities of BRD4, ER, HDAC1, P300, H3K27ac, Pol II public available ChIP-seq data of MCF7 separately treated with estrogen and tamoxifen; **(B)** bar graph of BRD4 ChIP-qPCR result on GREB1 promoter region of MCF7 separately treated with vehicle, fulvestrant(100nM), ZBC260(50nM) and Ful+ZBC260 for 48h; **(C, D)** bar graph of the RNA and protein level of GREB1 in MCF7 separately treated with vehicle, fulvestrant(100nM), ZBC260(50nM) and Ful+ZBC260 for 48h. * indicates p<0.05, ZBC260 compared to vehicle; # indicates p<0.05, Ful+ZBC260 compared to ZBC260, E2+ZBC260 compared to ZBC260 (mean ± SEM, n=3).

To investigate the epigenetic state of GREB1, we performed bioinformatics analysis of BRD4, ER, HDAC1, P300, H3K27ac, Pol II public available ChIP-seq datasets of MCF7 treated with estrogen and tamoxifen (**Table 1**). We find tamoxifen treatment induces less BRD4, ER and H3K27ac chromatin binding on the promoter regions of GREB1, whereas more HDAC1 binding events. What’s more, we find estrogen treatment induces more BRD4, ER, P300, H3K27ac and Pol II chromatin binding on the promoter regions of GREB1 (**Figure 8A**). Our ChIP-qPCR results demonstrate ZBC260 induce BRD4 less chromatin binding events on the promoter regions of GREB1, and ZBC260 combined with fulvestrant more impair the events, but estrogen rescue the events (**Figure 8B**). Our RT-qPCR and WB results also demonstrate ZBC260 induce the lower expression level of GREB1, and ZBC260 combined with fulvestrant induce the lowest expression level, but estrogen rescue the events (**Figures 8C, D**). In summary, this work reveals that BRD4 serves as a central epigenetic determinant for GREB1 expression, and its targeted inhibition can reprogram the chromatin landscape to override both estrogen and anti-estrogen signaling, offering a novel strategy to enhance therapeutic sensitivity.

## 4 Discussion

Despite the established efficacy of endocrine therapies in treating ERα-positive breast cancer, which accounts for about 75% of all cases, acquired resistance drives relapse in up to 30% of patients (7). Most cancers arise from complex genetic and epigenetic disturbances, and genetic alterations coincide with epigenetic reprogramming, which dynamically orchestrates the cellular programs underlying pathological manifestations (4). BRD4 is an epigenetic reader, and its chromatin occupancy on distal estrogen response elements (EREs) enriched for H3K27ac is required for recruitment and elongation of RNAPII on EREs in breast cancer. Multiple studies uncover BRD4 as a central regulator of ERα function and potential therapeutic target (8, 9). Our study reveals that the chromatin binding landscapes of H3K4ac, H3K9ac, H3K23ac, H3K27ac and BRD4 were significantly elevated in promoter regions in breast cancer, highlighting their potential as key epigenetic drivers in breast cancer pathogenesis. We further demonstrate that the transcriptional synergy between BRD4 and ER is not merely correlative but functional, driving a shared oncogenic program that can be effectively disrupted by targeting BRD4. Our ChIP-seq and RNA-seq analyses revealed that over 60% of ER promoter-binding events and 50% of its transcriptome overlap with that of BRD4. This substantial crosstalk encompasses crucial pathways such as the PI3K-Akt and estrogen signaling itself, which are frequently implicated in endocrinotherapy resistance. The efficacy of the BRD4-targeting PROTAC degrader, ZBC260, particularly enhances fulvestrant sensitivity, underscores the therapeutic potential of simultaneously disrupting both regulators. The superior performance of ZBC260 over the inhibitor JQ1 suggests that BRD4 protein degradation may be more effective than merely inhibiting its bromodomain.

A pivotal finding of our work is the identification of GREB1 as a primary node of convergence for BRD4 and ER signaling. While GREB1 is a well-established ER target gene, its regulation by a BRD4-dependent super-enhancer provides a novel mechanistic explanation for its robust expression. Our integrated multi-omics approach revealed extensive co-occupancy of BRD4 and ER on the GREB1 promoter and enhancer regions. The dynamic chromatin remodeling at this locus—where estrogen promotes the recruitment of BRD4, P300, and H3K27ac, while tamoxifen enriches for the repressor HDAC1—highlights its role as an epigenetic switch. The fact that combined BRD4 degradation and ER antagonism most potently suppressed GREB1 expression and protein levels confirms that both factors are non-redundant and essential for its full transcriptional output. Importantly, our findings directly link BRD4 to the clinical challenge of endocrine resistance. The poor prognosis associated with high BRD4 expression in patients treated with endocrine therapy, coupled with the upregulation of BRD4 in fulvestrant-resistant cell model, positions BRD4 as a biomarker and a driver of resistance. The identification of 58 genes coregulated by BRD4, ER, and implicated in resistance, with 12 being super-enhancer-associated, provides candidate targets for future investigation into novel combination therapies.

All mechanistic experiments in this study were performed in MCF7 cells, acquired endocrine resistance is a complex and heterogeneous process that can involve diverse molecular mechanisms, including ESR1 mutations, alternative growth factor receptor signaling (e.g., HER2, FGFR), and alterations in chromatin regulators beyond BRD4 (25, 26). Future studies using additional ER-positive endocrine therapy resistant derivatives (e.g., resistant cell lines, patient-derived xenografts, or organoids) will be necessary to determine the generalizability of our findings.

In conclusion, our data present a compelling model wherein BRD4 and ER cooperatively govern a critical transcriptional network, with GREB1 as a key effector, to promote breast cancer progression. The enhanced effect observed between BRD4 degradation and ER targeting offers a strong mechanistic rationale for a novel therapeutic strategy. BET degraders like ZBC260 in combination with standard endocrine therapy, particularly in patients with high BRD4 or GREB1 expression, are promising strategy for improved clinical outcomes.

## Data Availability

The datasets presented in this study can be found in online repositories. The names of the repository/repositories and accession number(s) can be found in the article/**Supplementary Material**.

## Supplementary material

Supplementary Table 1 The differential expression genes in MCF7 fulvestrant resistant cell line.

Supplementary Table 2 The annotation of MCF7 BRD4 and ER ChIP-seq datasets.

Supplementary Table 3 The differential expression genes in MCF7 separately treated with JQ1 and fulvestrant.

Supplementary Table 4 The annotation of enhancer and super-enhancer of MCF7 BRD4 ChIP-seq data.
